# Food and Drug Administration Expert Panel on Infant Formula “Operation Stork Speed” June 2025: Part 3, Marketing of Infant Formulas, Breastfeeding Support, Hypoallergenic Formulas, and Nutrition for Preterm Infants^☆^

**DOI:** 10.1016/j.advnut.2025.100585

**Published:** 2026-01-12

**Authors:** Steven A Abrams, James Thomas Brenna, Roger Clemens, Valeria C Cohran, Nan Du, Andrea Gilbaugh, Michael I Goran, Analeise Guild, John A Kerner, Thomas B Knudsen, Sushma Krishna, Timothy Sentongo

**Affiliations:** 1 Department of Pediatrics, Dell Medical School at the University of Texas, Austin, TX, United States; 2 Department of Regulatory and Quality Sciences, USC Mann School of Pharmacy and Pharmaceutical Sciences, University of Southern California, Los Angeles, CA, United States; 3 Department of Pediatrics, The Ann & Robert H. Lurie Children’s Hospital of Chicago, Feinberg School of Medicine, Chicago, IL, United States; 4 Center for Nutrition, Division of Gastroenterology, Hepatology, and Nutrition, Boston Children’s Hospital, Boston, MA, United States; 5 Clinical Nutrition, Stanford Medicine Children’s Health, Palo Alto, CA, United States; 6 Department of Pediatrics, Children’s Hospital Los Angeles and Keck School of Medicine, University of Southern California, Los Angeles, CA, United States; 7 Global Gastroschisis Foundation, United States; 8 Division of Gastroenterology, Hepatology, and Nutrition and Division of Neonatology and Developmental Medicine, Stanford University Medical Center, Palo Alto, CA, United States; 9 Department of Intelligent Systems Engineering, Indiana University, Bloomington, IN, United States; 10 Division of Neonatology, New York-Presbyterian–Weill Cornell Medicine, New York, NY, United States; 11 Department of Pediatrics, University of Chicago, Pritzker School of Medicine, Chicago, IL, United States

**Keywords:** infant formula, Operation Stork Speed, human milk, preterm infants, infant nutrition

## Abstract

The United States formula industry uses aggressive marketing strategies that exploit parental anxieties, undermine breastfeeding, and violate ethical international standards, all of which contribute to early formula introduction and disparities in breastfeeding. In addition, misleading or complex labeling contributes to caregiver and provider confusion regarding the content of formulas. A centralized, Food and Drug Administration–maintained database of infant formulas could support caregivers and healthcare professionals in countering misinformation and making evidence-based decisions. Human milk remains the reference standard for infant nutrition, with strong evidence supporting its role in reducing morbidity and mortality, especially in the neonatal intensive care unit (NICU). Despite this, disparities persist in breastfeeding rates, particularly among Black and Hispanic mothers. These are driven by structural barriers, including lack of paid parental leave and inadequate lactation support. Donor human milk (DHM) is the recommended alternative for high-risk infants in the NICU when the mother’s own milk is unavailable. However, due to lack of federal funding and oversight, high cost and inconsistent insurance coverage, the use of DHM is limited, especially in safety-net hospitals. Specialized hypoallergenic and metabolic formulas are essential for managing medical conditions including cow’s milk–protein allergy and inborn errors of metabolism. These are regulated as exempt formulas but must meet rigorous clinical standards. In the NICU, adequate early nutrition for preterm infants is crucial to reduce morbidity and mortality and improve long-term neurodevelopmental outcomes. Human milk fortifiers are composed of similar ingredients to infant formula and are used to improve the nutritional content of feedings given in the NICU and postdischarge. The use of live biotherapeutic products or probiotics as additives to preterm infants has been demonstrated in multiple studies to reduce the incidence of necrotizing enterocolitis, late-onset sepsis, and all-cause mortality.

**Statement of significance**

This manuscript provides information related to the marketing of infant formula and its use in special situations as provided at the Food and Drug Administration expert panel meeting in June 2025.

## Special Topics Related to Operation Stork Speed

Much of the focus on Operation Stork Speed, including the request for information from the Food and Drug Administration (FDA), has focused on nutrients and regulatory issues. However, other topics were discussed at the FDA expert panel meeting on 4 June, 2025, and are important in understanding the overall landscape of infant formula use in the United States [1]. In this paper, we consider these topics as they relate to infant formula and healthy infant nutrition in the United States.

## Marketing of Infant Formulas

Infant formula companies use many manipulative marketing strategies to prioritize profits and aggressive marketing conflicts with public health objectives for breastfeeding (Table 1). An investigation of infant formula products sold online in the United States found that companies are widely violating the WHO code (which is not accepted by the United States regulatory authorities) by using marketing that idealizes formula feeding and undermines breastfeeding [2]. Breastfeeding rates increased during the 2022 formula shortage in populations with historically higher reliance on infant formula, a finding that suggests the possibility of improving breastfeeding in these populations [3].

**TABLE 1:** Key issues and recommendations related to infant formula.

Topic	Key issues identified	Recommendations
Infant formula marketing	• Widespread marketing violations of the WHO code (not legally adopted in the United States) • Complex product labels with minimal guidance or standardization • Misleading claims (e.g., “brain building,” “immune support”) • Emotional manipulation of parental guilt/anxiety	• Enhance Food and Drug Administration regulation of marketing claims and digital advertising • Consider adopting WHO code elements to support breastfeeding and ensure truthful labeling
Caregiver and provider education	• Many caregivers unaware of clinical evidence or appropriate use • Health professionals often lack formal training in formula types • Social media and influencers (often sponsored) dominate information sharing	• Create Food and Drug Administration–maintained, evidence-based online formula comparison tool • Provide formal training to healthcare providers on infant formulas

Formula marketing may cause misconceptions about the benefits of formula use and can result in the early introduction of infant formula. Messages implying a benefit of infant formula compared with human milk are deceptive, as are claims that suggest product equivalence to human milk. In addition, product names and various health and nutrition claims can contribute to confusion for caregivers and healthcare professionals. Advertising may utilize emotional and psychological messaging; for example, 1 company represents its formula as “the one you feel proud to feed your baby.” Claims and even formula names may also target parental anxiety using terms such as “brain building,” “immune support,” “more efficient weight gain,” or “reduced crying within 24 h.” Products typically lack references for this type of claim, and when provided, citations often fail to support these claims; industry-funded trials also present a high risk of bias [4].

Marketing tactics used by the formula industry influence caregivers’ perceptions, as evidenced by a 2020 survey, which revealed that over half of infant caregivers believed infant formula can be better than human milk for digestion and brain development [5]. The authors also reported that some caregivers providing “toddler milk” to infants were unaware that it was not infant formula, demonstrating that cross-branding can cause confusion and may result in inappropriate products being offered to infants.

The labels of products marketed for toddlers aged >12 mo should clearly state that they are not appropriate for infants. The Committee on Nutrition of the American Academy of Pediatrics (AAP) has additionally recommended that these products not be positioned on store shelves in proximity to infant formulas as is currently common [6]. Enhanced regulatory oversight by the FDA for infant formula products and toddler “milk” marketing is needed to better protect infants and caregivers, perhaps by incorporating some aspects of the WHO Code of Marketing of Infant Formulas which also provides guidance related to toddler products [7].

Responsibility is often placed on caregivers to identify formulas that they believe are best for their infant. Pediatricians and other healthcare providers may give input, but currently much of the decision process occurs at the consumer level. Unfortunately, information is often highly shaped by formula marketing. It is difficult or impossible for families to determine the accuracy and relevance of the information provided online or from other advertising venues. Many parents are currently getting considerable additional formula-related information from online influencer sources such as “mommy groups” and blogs [8]. These spaces can also be influenced by infant formula manufacturers, without clear understanding or statements of conflicts of interest by those providing recommendations at these sites. Clear regulatory guidance about medical or other health claims provided on such sites would be helpful.

## Education

Product labels on infant formula products typically include long, complex ingredient lists, which can be difficult to understand. In addition, many healthcare providers, such as pediatricians and even neonatologists, may not receive standardized training on differences among infant formulas. A centralized, publicly accessible electronic database maintained by the FDA has been recommended to help with guidance in the event of another severe infant formula shortage [9]. A list of exempt formulas (specialized formulas that may not have nutrients at the levels mandated by the Infant Formula Act) is also publicly maintained by the FDA. However, no such list exists for routine (nonexempt) formulas. This type of formula database would be a valuable general educational resource for healthcare professionals and caregivers for navigating and comparing the many formula products on the market. In addition to listing formulas, this type of site should include information about where the products are produced and the nutrient contents, including bioactive ingredients. By providing impartial, science-backed information, this platform would enable families and healthcare professionals to better interpret and clarify the promotional messages from formula companies. Formulas could be organized by type (e.g., milk-based, partially hydrolyzed protein, and soy protein). Search filters based on infant health needs could be incorporated and translated into multiple languages to promote equitable access.

## Breast (Human) Milk

Maternal breast milk is considered the optimal reference standard for infant nutrition [10]. The components of breast milk include human milk oligosaccharides, growth factors, hormones, lipids, and other bioactive components that have been found to offer various protections, especially in premature infants, as discussed below [10]. Although the benefits of human milk have been well-documented, there are differences in mothers’ milk provision in Black and Hispanic mothers as compared with other groups [10] (Table 2). There are multiple obstacles, including structural, environmental, and individual factors, which may limit breastfeeding. Determinants may influence or dissuade mothers from breastfeeding, including cultural differences in acceptance of breastfeeding, limited lactation support, and the absence of paid maternity leave [11,12]. Strategies such as providing culturally matched peer counselors, communicating with families in a racially and culturally sensitive fashion, enhancing maternity leave and support for nursing in public may help increase breastfeeding rates [10].

**TABLE 2:** Key issues and recommendations on human milk and probiotics.

Topic	Key issues identified	Recommendations
Breastfeeding disparities	• Lower initiation and duration rates among Black and Hispanic mothers • Barriers include work pressures, lack of support/maternity leave, and historical mistrust	• Expand workplace protections and national paid leave • Enhance public breastfeeding infrastructure (e.g., lactation rooms) • Fund culturally tailored lactation support programs
DHM	• Milk banks are underfunded and reliant on public donations • Hospitals often absorb costs due to insurance gaps • Informal sharing is increasing	• Federally fund milk banks • Mandate insurance coverage for medically necessary DHM (e.g., California’s Donor Milk Law, 2025) • Public education campaigns on milk donation • Ensure safe outpatient access
Probiotics and live biotherapeutics	• Proven efficacy in reducing necrotizing enterocolitis, sepsis, and mortality in the preterm population • Routine use in Europe and Australia intensive care nurseries • 2023 Food and Drug Administration warning virtually halted United States use due to safety/quality concerns	• Prioritize development of Food and Drug Administration–regulated, pharmaceutical-grade products • Weigh risk/benefit before universal bans in intensive care nurseries

Abbreviation: DHM, donor human milk.

## Donor Human Milk

When mother’s own milk (MOM) is unavailable, the AAP recommends donor human milk (DHM) as the next-best option for very preterm infants and other high-risk neonates [13]. Public education about donor milk is limited, with some pediatricians not discussing it with their families [14]. In the United States, neonatal intensive care units (NICUs) typically obtain DHM from nonprofit milk banks, most of which are accredited by the Human Milk Banking Association of North America [15]. These banks recruit and screen volunteer donors, test milk for infectious diseases, pasteurize it for safety, and distribute it frozen to hospitals. Despite this well-established system, significant barriers limit DHM access and equitable usage nationwide. Because DHM is not consistently classified as a medical necessity, many public and private insurance plans do not provide coverage. As a result, hospitals, particularly those serving low-income communities, must often absorb the cost which can limit its use. In addition, human milk banks receive no dedicated federal funding and rely solely on public donations. Awareness of milk donation remains low due to a lack of large-scale public campaigns, as compared with blood donation, limiting donor recruitment and contributing to supply shortages. These shortages are exacerbated during public health emergencies, as seen during the 2022 United States infant formula crisis, when demand for DHM spiked [16].

To address these challenges, California implemented the Enhancing Access to DHM law in January 2025 [17]. This law mandates insurance coverage for medically necessary DHM and reduces some regulatory barriers, allowing more acute care hospitals to distribute DHM. These policy changes have already led to a 10% increase in DHM use in California NICUs over the first 6 mo of implementation. They are expected to improve health outcomes, reduce care costs, and advance health equity.

Outside the NICU, public demand for donor milk continues to grow. In some regions, pasteurized DHM is available for outpatient use when supplies allow, though costs are often prohibitive. National data on DHM use remain limited, but a 2021 survey found that 8% of >2000 respondents had used donor milk—about one-third of whom sourced it from unregulated channels [18]. In the absence of affordable, regulated access, some families turn to informal milk sharing networks or purchase milk online. Online communities and social media often influence these practices, shaping perceptions of professional medical advice [19]. Cultural and religious values may also influence decisions to engage in milk sharing, with some Muslim families hesitant to use DHM unless they have transparency about the donor [20]. However, informal milk sharing poses significant health and safety concerns due to the absence of regulatory oversight. Milk that has not undergone screening may carry risks of bacterial contamination or exposure to infectious diseases, medications, and other harmful substances [21]. Adulteration is also a documented issue: 1 United States study found cow’s milk in 10% of 102 human milk samples purchased anonymously online [22]. To protect infant health and ensure equitable access to safe DHM, a more robust and regulated national system—potentially under FDA oversight—is urgently needed.

## Hypoallergenic Formulas

There are 2 main types of hypoallergenic formulas currently available: extensively hydrolyzed formulas (EHFs) and amino acid-based formulas (AAFs). In EHFs, intact milk proteins are enzymatically broken down (hydrolyzed) into smaller peptides and free amino acids, which are less likely to trigger an allergic reaction. The casein hydrolysates used in these formulas have undergone extensive preclinical evaluation, including immunoassays, gel permeation chromatography, and guinea pig immunization studies. These assessments demonstrate that the resulting peptides are nonantigenic and have molecular weights <1200 Da. [23,24]. EHFs are generally indicated for infants with mild-to-moderate cow’s milk–protein allergy (CMPA). AAFs, also referred to as “elemental formulas,” contain proteins that have been completely broken down into free amino acids. These formulas are intended for infants with severe CMPA or multiple food allergies who do not tolerate EHF. AAFs are considered very unlikely to be allergenic because they contain no intact or partially hydrolyzed proteins. However, when possible, breastfeeding support should be continued in this situation.

Hypoallergenic formulas are classified as exempt infant formulas under FDA regulations. Exempt formulas are intended for infants with special medical or dietary needs, such as inborn errors of metabolism, cholestatic conditions, and allergies. Because hypoallergenic formulas are exempt from some compositional requirements that apply to routine infant formulas, but must still ensure safety and efficacy for their intended populations. Hypoallergenic formulas must comply with FDA nutrient specifications for infant formulas as outlined in 21 CFR 107.100 and must support normal growth and development. To be labeled hypoallergenic, these formulas must be supported by clinical evidence demonstrating tolerance in ≥90% of infants with confirmed allergies, with 95% confidence interval. This rigorous standard is established through randomized, double-blind, placebo-controlled trials, in accordance with AAP guidelines, which are recognized by the FDA. Currently, all hypoallergenic formulas available on the market have undergone such evaluations and meet these criteria [25]. Given their established clinical safety and efficacy, clinicians currently do not recommend any changes to these hypoallergenic formulas. It is crucial that the FDA clearly distinguishes that modifications or improvements made to standard infant formulas (e.g., limiting lactose or increasing specific nutrients) should not be automatically applied to exempt formulas, as even cow’s milk–protein intolerance does not imply lactose intolerance. Such changes should raise concerns among both clinicians and the infant formula industry.

## Issues Related to NICU and Long-Term Follow-up

The impact of early nutrition on outcomes in premature infants is well established [26,27]. Premature birth, particularly before 28 wk of gestation, interrupts critical stages of brain development [28]. Several studies have demonstrated that ensuring adequate caloric intake, particularly protein and energy balance, in extremely preterm infants can significantly enhance structural brain growth [29,30]. One study linked increased early protein intake with improved Bayley Mental Developmental Index scores in extremely preterm infants at 18 mo corrected age [31].

In postsurgical term neonates, optimal nutrition has been associated with shorter time to wound healing, reduced incidence of postoperative complications, and decreased length of hospital stay [32,33]. Early optimal nutrition minimizes the severity of neonatal illnesses such as bronchopulmonary dysplasia, necrotizing enterocolitis (NEC), and late-onset sepsis [34]. NEC is the leading gastrointestinal emergency in very low birth weight infants, with mortality rates as high as 30% [35]. Human milk is the only intervention proven to reduce the risk of NEC [36]. Prematurity and its complications result in substantial healthcare costs, ∼$332,000, compared with $7247 for a term infant [37]. Prior studies demonstrated that the delivery of MOM is associated with decreased costs of morbidities. Johnson et al. [38] reported reduced incidence of NEC and hospital costs with infants who received MOM and DHM as compared with those receiving MOM and formula.

For most neonates in the NICU, the AAP recommends the use of exclusive human milk as the reference standard for nutrition [39]. The numerous benefits of human milk, especially the immunoprotective properties, are well established and are linked to reducing the risk of NEC, sepsis, and mortality. Studies have also demonstrated reduced risk of postdischarge growth failure as well as improved long-term brain development and neurodevelopmental outcome in infants fed human milk in the NICU [40,41].

However, for the extremely preterm infant population, human milk alone is often insufficient to meet the higher caloric and nutritional demands [42]. For these patients, human milk is frequently supplemented or “fortified” with the addition of human milk fortifier (HMF). HMF is a concentrated liquid or powder derived from either bovine milk or human donor milk. It is composed of a concentrated form of carbohydrates, lipids, and proteins along with added minerals and micronutrients [43,44]. More studies are needed to evaluate the benefits specific to the use of human milk compared with bovine-derived fortifier [45].

Infant formula plays an important role in NICU nutrition when maternal breast milk is unavailable, insufficient, or sometimes contraindicated. Infant formula is commonly used as a supplement to maternal milk in late preterm and term infants and/or as part of a discharge regimen to support catch-up growth in extremely preterm infants [46]. Infants who have unique medical needs, such as gastrointestinal anomalies, metabolic disorders, and postsurgical complications, require specialized formulas. For example, infants with CMPA or neonates with intestinal failure, such as associated with short bowel syndrome, often require extensively hydrolyzed protein or AAFs. Infants with inborn errors of metabolism, such as phenylketonuria or maple syrup urine disease, require metabolic formulas that exclude specific amino acids [47,48].

The use of live biotherapeutic products (LBPs) or probiotics as additives to human milk or infant formula has been demonstrated in multiple studies to reduce the incidence of NEC, late-onset sepsis, and all-cause mortality [49]. Probiotic strains from Bifidobacterium and Lactobacillus geneses reduce preterm intestinal inflammation, promote healthy gut microbiota, and are safely utilized in NICUs throughout Europe and Australia. However, in October 2023, the FDA issued a warning against the use of probiotics in preterm infants in the United States, due to concerns that commercially available products are not subject to the same rigorous safety and quality standards as drugs or biologics and may therefore pose risks to this vulnerable population. Following this advisory, many NICUs in the United States discontinued the use of probiotics, raising concerns of the potential for increases in NEC and mortality. Studies using pharmaceutical-grade LBPs are underway to evaluate the safety and outcomes in the very preterm population [50].

In conclusion, marketing strategies from formula companies can be misleading, exploit parental anxieties, and contribute to confusion among caregivers and providers (Table 1). Stricter regulations should be enacted to protect families and promote informed feeding decisions. Additionally, healthcare providers and parents often lack access to reliable information about formula products. A centralized FDA-maintained database could address this gap and provide clarity on messaging from manufacturers.

Hypoallergenic infant formulas include EHFs for mild-to-moderate CMPA and AAFs for severe CMPA or multiple food allergies. Classified as exempt formulas under FDA regulations, they must meet strict clinical standards to be labeled hypoallergenic and should not be subject to modifications intended for standard formulas without thorough evaluation.

Racial and socioeconomic disparities disproportionately affect breastfeeding rates, particularly among Black and Hispanic mothers (Table 2). Systemic reforms are needed to address structural barriers such as a lack of paid maternity leave and inadequate lactation support. DHM is the AAP-recommended alternative to a MOM, especially for premature infants, and has been demonstrated to reduce morbidity and mortality in this population. However, DHM remains inaccessible to many NICUs due to insurance coverage gaps, lack of funding, and limited public awareness regarding donation.

Early optimal nutrition for preterm infants in the NICU improves long-term neurodevelopmental outcomes and reduces complications such as NEC. Although human milk is the safest, it often requires supplementation with bovine or human-derived fortifiers. Specialized formulas also play an important role for infants with GI or surgical complications and those with metabolic disorders. In addition, although research supports the use of probiotics to reduce NEC, a 2023 FDA warning has led to their removal from United States NICUs.

## Author contributions

The authors’ responsibilities were as follows – SAA, JTB, RC, VCC, ND, AG, MIG, AG, JAK, TBK, SK, TS: participated in the conference described and contributed to the preparation of the manuscript; and all authors: read and approved the final manuscript.

## Funding

JTB has received honoraria or travel funds from the Global Organization for EPA and DHA, the global dairy platform, Danone, and research support from the National Cattlemen’s Beef Association. The authors have no additional financial support to declare.

## Conflict of interest

JTB has received honoraria or travel funds from the Global Organization for EPA and DHA, the global dairy platform, Danone, and research support from the National Cattlemen’s Beef Association. MIG has previously served as a scientific advisor for Begin Health and Bobbi. VCC has served on the Speaker’s Bureau for Nutricia and Abbott Nutrition. SAA is Editor-in-Chief of *Advances in Nutrition* and played no role in the journal’s evaluation of the manuscript. Other authors declare that they have no known competing financial interests or personal relationships that could have appeared to influence the work reported in this paper.
